# Multi-Faceted Post-Transcriptional Functions of HIV-1 Rev

**DOI:** 10.3390/biology1020165

**Published:** 2012-07-20

**Authors:** Kuan-Teh Jeang

**Affiliations:** Molecular Virology Section, Laboratory of Molecular Microbiology, National Institutes of Allergy and Infectious Diseases, the National Institutes of Health, Bethesda, MD 20892, USA; Email: kj7e@nih.gov; Tel.: +1-301-496-6680; Fax: +1-301-480-3686

**Keywords:** Rev, CRM1, DDX3, RNA export, RNA translation, RNA packaging

## Abstract

Post-transcriptional regulation of HIV-1 gene expression is largely governed by the activities of the viral Rev protein. In this minireview, the multiple post-transcriptional activities of Rev in the export of partially spliced and unspliced HIV-1 RNAs from the nucleus to the cytoplasm, in the translation of HIV-1 transcripts, and in the packaging of viral genomic RNAs are reviewed in brief.

## 1. Introduction

**Figure 1:**
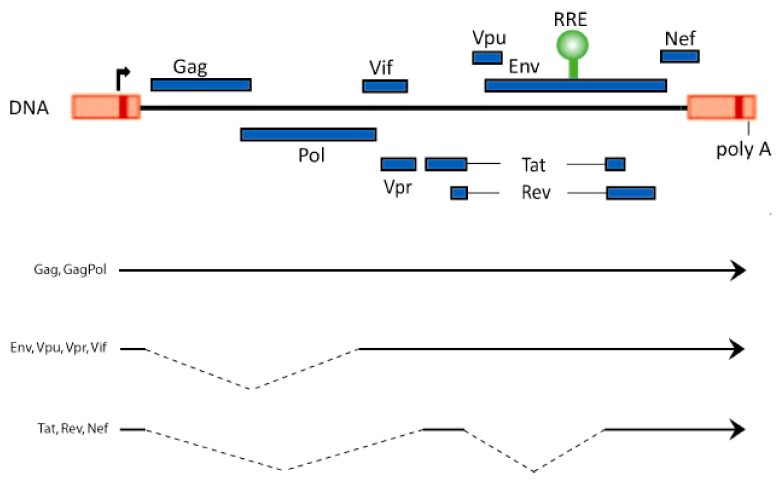
Schematic representation of the HIV-1 genome and its unspliced and spliced RNAs. The open reading frames and the position of the Rev‑responsive element (RRE; green) are shown on top. The bottom lines represent the 9 kb unspliced, the 4 kb singly spliced, and the 1.8 kb doubly spliced HIV-1 RNAs.

The genome of the human immunodeficiency virus, HIV-1, encodes three classes of viral transcripts: 9 kb unspliced RNA, 4 kb partially spliced RNA, and 1.8 kb fully spliced RNA. The 9 kb RNA codes for Gag and GagPol proteins, while the 4 kb RNA generates the Env, Vif, Vpr, and Vpu proteins, and the 1.8 kb moiety produces Rev, Tat, and Nef (Figure 1). Full length HIV-1 RNA contains at least 5 splice donor sites and 9 splice acceptor sites whose permutations and combinations lead to the generation of approximately 40 distinct viral mRNAs [1,2,3]. 

HIV-1 splicing generally proceeds sub-optimally [4,5]. A number of host cell proteins including arginine/serine rich (SR) proteins [SC35, ASF/SF2 (alternative splicing factor/splicing factor 2), SRp40 (SR protein 40), and 9G8] and heterogenous ribonucleoproteins (hnRNPs; hnRNP A1, hnRNP A2, and hnRNP A3) have been reported to influence splicing as well as the fate of unspliced/spliced HIV-1 mRNAs [3,6,7,8]. While the HIV-1 Tat protein drives viral transcription [9,10,11], the Rev protein governs multifaceted aspects of post-transcriptional expression and utilization of viral RNAs. 

Rev is a 116 amino acid protein. Like Tat, Rev is an RNA-binding protein containing a stretch of highly basic arginine residues. While Tat binds to the ~80 nucleotide TAR RNA hairpin motif which is contained in all classes of HIV-1 RNAs, Rev binds to the ~350 nucleotide stem-loop-rich RRE (Rev‑responsive element) RNA motif (position 7709 to 8063; Figure 1) which is present in 9 kb, 4 kb, but not 1.8 kb viral transcripts. A key understanding of Rev function has emerged from the characterization of its arginine-rich nuclear localization (NLS) and its leucine-rich nuclear export (NES) signals. Currently, it is understood that Rev is a nuclear-cytoplasmic shuttling protein. It is ferried into the nucleus in an energy-dependent manner by NLS-interaction with importin b or other nuclear import factors, and it exits the nucleus via NES-engagement with the CRM1 protein [12,13,14].

Four recent papers have provided new structural insights into Rev-RRE interaction [15,16,17,18]. Previously, it was reported that Rev nucleates at its primary RRE-binding site as a monomer [19], although multiple molecules of Rev assembled on the RRE are needed for function [15]. The new studies indicate that at least 6 Rev monomers converge on the RRE with the subsequent formation of RRE-RNA-Rev dimers that then further oligomerize into larger RNP-complexes. Within the RRE‑RNA-Rev dimer, the two Rev protein molecules form a V shape through protein-protein contact via hydrophobic residues at amino acid positions 12, 16, and 60. This V shaped protein-dimer positions the respective arginine-rich RNA binding helicase from each Rev monomer in a way that each presents a different contact surface to cooperatively engage the RRE-containing RNA. These new studies offer elegant structural insights, but still leave unanswered how the Rev-RRE structure shapes the various Rev biological activities. 

## 2. Rev-Dependent Export of Partially Spliced and Unspliced HIV-1 RNAs from the Nucleus

**Figure 2:**
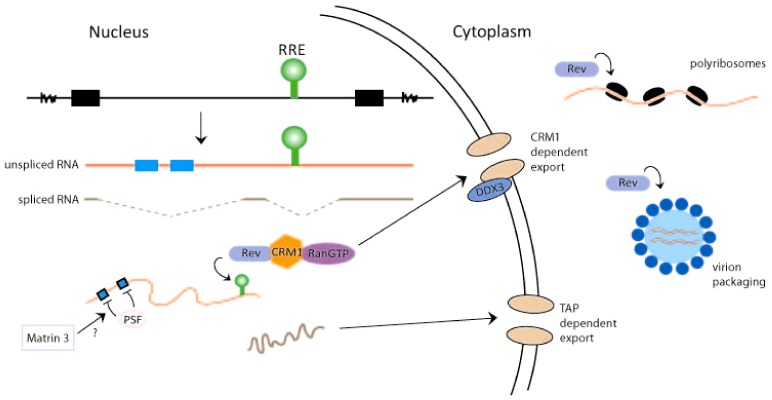
Rev activities influence HIV-1 RNA transport, translation, and packaging. The provirus is shown to produce unspliced RNA (with RRE, green, and instability sequence elements (INS), blue) and fully spliced HIV-1 RNA (with no RRE or INS sequences). The export of unspliced (red)/partially spliced HIV-1 RNA through a CRM1 dependent pathway and the export of fully spliced viral RNA (brown) through a TAP dependent pathway are shown. In the model, negative nuclear factor(s) that binds the INS sequence (e.g., PSF) and participates in the retention of unspliced/partially spliced viral RNAs in the nucleus is illustrated. Rev/CRM1/RanGTP and Matrin 3 are considered to cooperate with DDX3 RNA helicase for the export of unspliced/partially spliced HIV-1 RNAs from the nucleus through the nuclear pores. In the cytoplasm, Rev enhances viral RNA translation on polyribosomes and its packaging into virion particles. The exact cellular factors that assist Rev in these latter functions remain to be identified and characterized.

Eukaryotic cells typically retain unspliced and incompletely spliced RNAs in the nucleus while allowing intron-less, fully-spliced, RNAs to exit the nucleus. Because retroviruses use unspliced full‑length 9 kb viral RNA as the genomic RNA for progeny virions, as well as to encode Gag and GagPol proteins, and partially spliced 4 kb RNA (in the case of HIV-1) to synthesize Env, Vif, Vpr, and Vpu proteins, these viruses must develop mechanism(s) that actively release such RNAs from the nucleus into the cytoplasm. Retroviruses achieve this function through two major means. Genetically simpler retroviruses like Mason-Pfizer monkey virus (MPMV) have a *cis*-RNA sequence, the constitutive transport element (CTE), that exists in unspliced/partially spliced viral RNAs [20]. CTE‑containing RNAs are recognized by the NFX1 (nuclear transcription factor X-box binding 1)/TAP (tyrosine kinase interacting protein associated protein) transport factor (which is normally used for the export of spliced cellular transcripts) for egress out of the nucleus [21,22,23] (Figure 2). Genetically complex retroviruses like HIV-1 have, instead of a CTE, an RRE-RNA sequence that is a binding-target for a viral protein (*i.e.*, Rev). Rev binds RRE-RNA and docks it to the cellular export protein, CRM1 (chromosome region maintenance 1) [24,25]. The RRE-RNA-Rev-CRM1 complex is then transported together from the nucleus into the cytoplasm (Figure 2). 

It should be emphasized that besides the RRE-sequence, unspliced and partially spliced HIV-1 RNAs contain several short instability sequence elements (INS) [26,27]. While RRE is a binding site for “positive” RNA-transport factors (Rev and CRM1), the INS elements are thought to be sites for “negative” nuclear retention factors that sequester INS-containing RNAs in the nucleus. The importance of INS sequences for the nuclear retention of RRE-RNA-containing transcripts was demonstrated by mutagenesis studies that changed the INS nucleotide sequences in a manner to optimize their translation codon-usage. These sequence changes are thought to free the INS motifs from being bound and sequestered by nuclear retention factors rendering RRE/INS containing RNAs to become constitutively competent, in a Rev-independent manner, for nuclear-cytoplasmic export [26,27]. Currently, the identities of the negative factors that putatively retain HIV-1 INS‑containing sequences in the nucleus are not fully clear. The list of proteins that can bind HIV-1 INS elements include hnRNP A1 [28], polypyrimidine tract binding protein (PTB) [28], polypyrimidine tract binding protein-associated splicing factor (PSF) [29] and poly(A)-binding protein (PABP) [30]. Some investigators have suggested that the PSF (PTBP-associated splicing factor) protein might be a major nuclear retention/destabilization factor for unspliced/partially spliced HIV-1 transcripts [29]. Intriguingly, two independent laboratories have reported recently that a PSF‑associated factor, Matrin 3, acts in a Rev-dependent fashion to increase the cytoplasmic expression of RRE‑containing transcripts [31,32]. The results suggest that Matrin 3 may act, in part, by countering the nuclear retention/destabilization of RRE/INS-containing RNAs by PSF (Figure 2). 

In addition to CRM1, Rev—RRE RNA interaction relevant to nuclear-cytoplasmic transport likely involves several other host cell co-factors. These include Ran (Ras-related nuclear protein), FG (phenylalanine-glycine)-repeat nucleoporins, RIP (Rev-interacting protein)/RAB (Rev/Rex activation domain-binding protein) [33,34], and a multitude of RNA helicases such as DDX3 [DEAD (Asp-Glu-Ala-Asp) box 3] [35], DDX1 [36,37], and RNA helicase A (RHA) [38]. Of interest, HIV-1 infection is known to up regulate the cellular expression of several RNA helicases including DHX9, DDX11, DDX18, DDX21 and DDX24 [39], and a recent mass spectrometry-based proteomic study identified the binding to Rev-RRE-RNA of eight cellular RNA helicases, DDX1, DDX3, DDX5, DHX9 (RHA), DDX17, DDX24, DHX36, and DDX47 [40]. How these helicases act in stabilizing, transporting, localizing, and perhaps translating RRE-containing HIV-1 RNAs remains to be clarified. 

The RNA-cap structure appears to be an additional component in the regulated transport of HIV-1 unspliced/partially spliced RNAs from the nucleus to the cytoplasm. The human cellular RNA methyltransferase, PIMT (peroxisome proliferator-activated protein with methyltransferase domain), was recently shown to enhance Rev-mediated export of HIV-1 RNA [41]. PIMT is an RNA methyl transferase that selectively hypermethylates the monomethylated m7G RNA-cap to a trimethylated TMG-cap on unspliced/partially, but not fully, -spliced HIV-1 transcripts. The selectivity of hypermethylation occurs because Rev recruits PIMT to RRE-containing RNAs. TMG-capped RNAs, like snRNAs [42] and snoRNAs [43], are substrates for recognition by the CRM1 transporter protein, and it was suggested that TMG-capped HIV-1 RNAs might accordingly be a *cis*-RNA element specifying CRM1-mediated transport from the nucleus into the cytoplasm. 

## 3. Rev and the Translation of HIV-1 RNA

Several early reports identified a role for Rev in promoting the translation of RRE-containing HIV‑1 mRNAs [44,45]. Molecular evidence showed that independent of its activity in exporting RRE-containing RNA from the nucleus, Rev enhances the translation of RRE-containing RNAs such as Gag by approximately 100 fold. Specifically, it was reported that Rev increased the association of Gag, Vif, Vpr, Vpu/Env RNAs with the translating polyribosomes [45] (Figure 2). How Rev achieves this result is not known. In a recent proteomic study, Rev was curiously found to associate with several mitochondrial (MRPS9, MRPS28, MRPL42; [40]), but no cytoplasmic, ribosomal proteins. Hence, a direct Rev effect on polyribosome components seems an unlikely mechanistic explanation for increased translation. Potentially, Rev interaction with cofactors such as Sam68 [46,47] and RHA/DHX9 [48] may reconfigure RRE-containing RNA for their targeting to and association with the cytoplasmic translational machinery. Additionally, Rev could directly interact with the 5′ UTR of HIV-1 RNAs to influence translation [49]. Additionally, the observed increase in translation may be secondary to Rev stabilization of RRE/INS-RNAs. In such view, enhanced translation is a consequence of increased RNA stability which could be achieved in RRE- and INS-containing RNAs by codon‑optimized silent mutations in the INS sequences or by providing the Rev protein in *trans* [27].

For HIV-1 and other retroviral RNAs (e.g., MMTV, [50]), an emerging picture is that nuclear pathways and cofactors used to export the viral RNA from the nucleus into the cytoplasm ultimately influence the localization and translational efficiency of the RNA in the cytoplasm. Rev interaction with RRE (and INS)—containing RNAs in the nucleus affects the removal of negative nuclear impediments as well as conferring positive effects to the cytoplasmic translation of RNAs. The exact balancing mechanistic details remain unclear and are unlikely to become more clear until additional relevant factors are identified and characterized. 

## 4. Rev and HIV-1 RNA Packaging

The full length unspliced HIV-1 RNA encodes the Gag-Pol proteins, but it is also the genomic RNA packaged into virions. A number of events impact the packaging of genomic viral RNA into virions. These include the appropriate exit from the nucleus of unspliced HIV-1 RNA, the dimerization of the genomic RNA, and Gag-RNA interaction in the cytoplasm prior to particle assembly and budding at the plasma membrane. As discussed above, Rev is pivotally important for the nuclear export of unspliced full length HIV-1 RNA from the nucleus. Recently a new activity for Rev/RRE in the packaging of genomic HIV-1 RNA has been broached. 

It is conventionally held that the *cis* 5' untranslated region (UTR) ~120 bp (ψ) signal [51] is important for packaging; however, recent data have shown that Rev/RRE plays a role in viral RNA encapsidation and influences HIV-1 infectivity by more than 1,000 fold [52,53,54,55]. Because the Rev protein is not found in virions, it is unlikely to contribute a direct role in the assembly of RRE‑containing RNAs into viral particles. Interestingly, the packaging activity of Rev correlates in part with the nuclear export of RNA suggesting that this activity of Rev is indirectly influenced by the nuclear events (e.g., RNP ribonuclear protein assembly) experienced by RRE containing RNAs [52,53]. For example, Rev and RRE dependent viral RNA encapsidation was 100 fold higher than similar viral RNA engineered to be Rev and RRE-independent by INS codon optimization of Gag RNA or by substituting CTE in place of RRE. The interpretation of these results is that Rev/RRE RNAs transit a nuclear export pathway different from that used by codon optimized Gag RNAs or CTE-containing viral RNAs [23,54], suggesting that the paths experienced by the RNAs matter in how the RNAs will ultimately be packaged into virions. Moreover, because the provision of Rev alone is not sufficient for the efficient encapsidation of HIV-1 RNA that is engineered to be exported via a CRM1 independent pathway, the findings implicate that CRM1/Rev—RRE RNA interaction is also critical for optimal HIV-1 RNA encapsidation. One should also keep in mind that full length unspliced viral RNA that is directed to the polyribosome for translation competes with its use for virion packaging. Thus, Rev activity that affects the translation of RRE-RNA also indirectly influences RNA-packaging into virions [56]. 

## 5. Concluding Remarks

To date, the study of HIV‐1 Rev has contributed to our understanding of intricate RNA-protein interactions that underlie RNA transport, translation and virion packaging. What was once considered as perhaps three discrete events now appear to be fully connected. Which pathway an RRE-containing RNA uses to exit the nucleus is apparently consequential to how it is destined for translation and packaging. If that RNA enters the polyribosome for translation then such choice excludes its utility for virion packaging. In the overall picture, Rev interaction with different cellular cofactors is also important in orchestrating the interconnected balance between roles in transport, translation, or packaging.

## References

[1] Purcell D.F., Martin M.A. (1993). Alternative splicing of human immunodeficiency virus type 1 mRNA modulates viral protein expression, replication, and infectivity. J. Virol..

[2] McLaren M., Marsh K., Cochrane A. (2008). Modulating HIV-1 RNA processing and utilization. Front. Biosci..

[3] Exline C.M., Feng Z., Stoltzfus C.M. (2008). Negative and positive mRNA splicing elements act competitively to regulate human immunodeficiency virus type 1 vif gene expression. J. Virol..

[4] Stoltzfus C.M., Madsen J.M. (2006). Role of viral splicing elements and cellular RNA binding proteins in regulation of HIV-1 alternative RNA splicing. Curr. HIV. Res..

[5] Kammler S., Otte M., Hauber I., Kjems J., Hauber J., Schaal H. (2006). The strength of the HIV-1 3' splice sites affects Rev function. Retrovirology.

[6] Cochrane A.W., McNally M.T., Mouland A.J. (2006). The retrovirus RNA trafficking granule: From birth to maturity. Retrovirology.

[7] Bolinger C., Boris-Lawrie K. (2009). Mechanisms employed by retroviruses to exploit host factors for translational control of a complicated proteome. Retrovirology.

[8] Saliou J.M., Bourgeois C.F., yadi-Ben M.L., Ropers D., Jacquenet S., Marchand V., Stevenin J., Branlant C. (2009). Role of RNA structure and protein factors in the control of HIV-1 splicing. Front. Biosci..

[9] Berkhout B., Silverman R.H., Jeang K.T. (1989). Tat trans-activates the human immunodeficiency virus through a nascent RNA target. Cell.

[10] Lever A.M., Jeang K.T. (2011). Insights into cellular factors that regulate HIV-1 replication in human cells. Biochemistry.

[11] Nekhai S., Jeang K.T. (2006). Transcriptional and post-transcriptional regulation of HIV-1 gene expression: Role of cellular factors for Tat and Rev. Future Microbiol..

[12] Lever A.M., Jeang K.T. (2006). Replication of human immunodeficiency virus type 1 from entry to exit. Int. J. Hematol..

[13] Shida H. (2012). Role of Nucleocytoplasmic RNA transport during the life cycle of retroviruses. Front. Microbiol..

[14] Okada H., Zhang X., Ben F.I., Nagai M., Suzuki H., Ohashi T., Shida H. (2009). Synergistic effect of human CycT1 and CRM1 on HIV-1 propagation in rat T cells and macrophages. Retrovirology.

[15] Daugherty M.D., D'Orso I., Frankel A.D. (2008). A solution to limited genomic capacity: Using adaptable binding surfaces to assemble the functional HIV Rev oligomer on RNA. Mol. Cell.

[16] Daugherty M.D., Booth D.S., Jayaraman B., Cheng Y., Frankel A.D. (2010). HIV Rev response element (RRE) directs assembly of the Rev homooligomer into discrete asymmetric complexes. Proc. Natl. Acad. Sci. USA.

[17] Daugherty M.D., Liu B., Frankel A.D. (2010). Structural basis for cooperative RNA binding and export complex assembly by HIV Rev. Nat. Struct. Mol. Biol..

[18] DiMattia M.A., Watts N.R., Stahl S.J., Rader C., Wingfield P.T., Stuart D.I., Steven A.C., Grimes J.M. (2010). Implications of the HIV-1 Rev dimer structure at 3.2 A resolution for multimeric binding to the Rev response element. Proc. Natl. Acad. Sci. USA.

[19] Pond S.J., Ridgeway W.K., Robertson R., Wang J., Millar D.P. (2009). HIV-1 Rev protein assembles on viral RNA one molecule at a time. Proc. Natl. Acad. Sci. USA.

[20] Bray M., Prasad S., Dubay J.W., Hunter E., Jeang K.T., Rekosh D., Hammarskjold M.L. (1994). A small element from the Mason-Pfizer monkey virus genome makes human immunodeficiency virus type 1 expression and replication Rev-independent. Proc. Natl. Acad. Sci. USA.

[21] Braun I.C., Rohrbach E., Schmitt C., Izaurralde E. (1999). TAP binds to the constitutive transport element (CTE) through a novel RNA-binding motif that is sufficient to promote CTE-dependent RNA export from the nucleus. EMBO J..

[22] Zolotukhin A.S., Michalowski D., Smulevitch S., Felber B.K. (2001). Retroviral constitutive transport element evolved from cellular TAP(NXF1)-binding sequences. J. Virol..

[23] Gruter P., Tabernero C., von K.C., Schmitt C., Saavedra C., Bachi A., Wilm M., Felber B.K., Izaurralde E. (1998). TAP, the human homolog of Mex67p, mediates CTE-dependent RNA export from the nucleus. Mol. Cell.

[24] Fornerod M., Ohno M., Yoshida M., Mattaj I.W. (1997). CRM1 is an export receptor for leucine-rich nuclear export signals. Cell.

[25] Hakata Y., Yamada M., Mabuchi N., Shida H. (2002). The carboxy-terminal region of the human immunodeficiency virus type 1 protein Rev has multiple roles in mediating CRM1-related Rev functions. J. Virol..

[26] Schwartz S., Campbell M., Nasioulas G., Harrison J., Felber B.K., Pavlakis G.N. (1992). Mutational inactivation of an inhibitory sequence in human immunodeficiency virus type 1 results in Rev-independent gag expression. J. Virol..

[27] Schneider R., Campbell M., Nasioulas G., Felber B.K., Pavlakis G.N. (1997). Inactivation of the human immunodeficiency virus type 1 inhibitory elements allows Rev-independent expression of Gag and Gag/protease and particle formation. J. Virol..

[28] Black A.C., Luo J., Chun S., Bakker A., Fraser J.K., Rosenblatt J.D. (1996). Specific binding of polypyrimidine tract binding protein and hnRNP A1 to HIV-1 CRS elements. Virus Genes.

[29] Zolotukhin A.S., Michalowski D., Bear J., Smulevitch S.V., Traish A.M., Peng R., Patton J., Shatsky I.N., Felber B.K. (2003). PSF acts through the human immunodeficiency virus type 1 mRNA instability elements to regulate virus expression. Mol. Cell. Biol..

[30] Afonina E., Neumann M., Pavlakis G.N. (1997). Preferential binding of poly(A)-binding protein 1 to an inhibitory RNA element in the human immunodeficiency virus type 1 gag mRNA. J. Biol. Chem..

[31] Kula A., Guerra J., Knezevich A., Kleva D., Myers M.P., Marcello A. (2011). Characterization of the HIV-1 RNA associated proteome identifies Matrin 3 as a nuclear cofactor of Rev function. Retrovirology.

[32] Yedavalli V.S., Jeang K.T. (2011). Matrin 3 is a co-factor for HIV-1 Rev in regulating post-transcriptional viral gene expression. Retrovirology.

[33] Neville M., Stutz F., Lee L., Davis L.I., Rosbash M. (1997). The importin-beta family member Crm1p bridges the interaction between Rev and the nuclear pore complex during nuclear export. Curr. Biol..

[34] Jones T., Sheer D., Bevec D., Kappel B., Hauber J., Steinkasserer A. (1997). The human HIV-1 Rev binding-protein hRIP/Rab (HRB) maps to chromosome 2q36. Genomics.

[35] Yedavalli V.S., Neuveut C., Chi Y.H., Kleiman L., Jeang K.T. (2004). Requirement of DDX3 DEAD box RNA helicase for HIV-1 Rev-RRE export function. Cell.

[36] Fang J., Kubota S., Yang B., Zhou N., Zhang H., Godbout R., Pomerantz R.J. (2004). A DEAD box protein facilitates HIV-1 replication as a cellular co-factor of Rev. Virology.

[37] Edgcomb S.P., Carmel A.B., Naji S., mbrus-Aikelin G., Reyes J.R., Saphire A.C., Gerace L., Williamson J.R. (2012). DDX1 is an RNA-dependent ATPase involved in HIV-1 Rev function and virus replication. J. Mol. Biol..

[38] Li J., Tang H., Mullen T.M., Westberg C., Reddy T.R., Rose D.W., Wong-Staal F. (1999). A role for RNA helicase A in post-transcriptional regulation of HIV type 1. Proc. Natl. Acad. Sci. USA.

[39] Krishnan V., Zeichner S.L. (2004). Alterations in the expression of DEAD-box and other RNA binding proteins during HIV-1 replication. Retrovirology.

[40] Naji S., Ambrus G., Cimermancic P., Reyes J.R., Johnson J.R., Filbrandt R., Huber M.D., Vesely P., Krogan N.J., Yates J.R. (2012). Host cell interactome of HIV-1 Rev includes RNA helicases involved in multiple facets of virus production. Mol. Cell. Proteomics.

[41] Yedavalli V.S., Jeang K.T. (2010). Trimethylguanosine capping selectively promotes expression of Rev-dependent HIV-1 RNAs. Proc. Natl. Acad. Sci. USA.

[42] Plessel G., Fischer U., Luhrmann R. (1994). m3G cap hypermethylation of U1 small nuclear ribonucleoprotein (snRNP) *in vitro*: Evidence that the U1 small nuclear RNA-(guanosine-N2)-methyltransferase is a non-snRNP cytoplasmic protein that requires a binding site on the Sm core domain. Mol. Cell. Biol..

[43] Terns M.P., Grimm C., Lund E., Dahlberg J.E. (1995). A common maturation pathway for small nucleolar RNAs. EMBO J..

[44] Arrigo S.J., Chen I.S. (1991). Rev is necessary for translation but not cytoplasmic accumulation of HIV-1 vif, vpr, and env/vpu 2 RNAs. Genes Dev..

[45] D'Agostino D.M., Felber B.K., Harrison J.E., Pavlakis G.N. (1992). The Rev protein of human immunodeficiency virus type 1 promotes polysomal association and translation of gag/pol and vpu/env mRNAs. Mol. Cell. Biol..

[46] Suhasini M., Reddy T.R. (2009). Cellular proteins and HIV-1 Rev function. Curr. HIV Res..

[47] He J.J., Henao-Mejia J., Liu Y. (2009). Sam68 functions in nuclear export and translation of HIV-1 RNA. RNA Biol..

[48] Hartman T.R., Qian S., Bolinger C., Fernandez S., Schoenberg D.R., Boris-Lawrie K. (2006). RNA helicase A is necessary for translation of selected messenger RNAs. Nat. Struct. Mol. Biol..

[49] Groom H.C., Anderson E.C., Dangerfield J.A., Lever A.M. (2009). Rev regulates translation of human immunodeficiency virus type 1 RNAs. J. Gen. Virol..

[50] Boeras I., Sakalian M., West J.T. (2012). Translation of MMTV Gag requires nuclear events involving splicing motifs in addition to the viral Rem protein and RmRE. Retrovirology.

[51] Berkowitz R.D., Hammarskjold M.L., Helga-Maria C., Rekosh D., Goff S.P. (1995). 5' regions of HIV-1 RNAs are not sufficient for encapsidation: Implications for the HIV-1 packaging signal. Virology.

[52] Brandt S., Blissenbach M., Grewe B., Konietzny R., Grunwald T., Uberla K. (2007). Rev proteins of human and simian immunodeficiency virus enhance RNA encapsidation. PLoS Pathog..

[53] Blissenbach M., Grewe B., Hoffmann B., Brandt S., Uberla K. (2010). Nuclear RNA export and packaging functions of HIV-1 Rev revisited. J. Virol..

[54] Cochrane A. (2009). How does the journey affect the message(RNA)?. RNA Biol..

[55] Cockrell A.S., van P.H., Santistevan N., Ma H., Kafri T. (2011). The HIV-1 Rev/RRE system is required for HIV-1 5' UTR cis elements to augment encapsidation of heterologous RNA into HIV-1 viral particles. Retrovirology.

[56] Lever A.M. (2007). HIV-1 RNA packaging. Adv. Pharmacol..

